# Autotrophic methylotrophy with no methanol dehydrogenase (MDH) in a strain of fluorescent *Pseudomonas*

**DOI:** 10.7717/peerj.20614

**Published:** 2026-02-02

**Authors:** Paolo De Marco

**Affiliations:** University Institute of Health Sciences (1H-TOXRUN, IUCS-CESPU), UCIBIO - Applied Molecular Biosciences Unit, Gandra, Portugal; University Institute of Health Sciences - CESPU, Associate Laboratory i4HB - Institute for Health and Bioeconomy, Gandra, Portugal

**Keywords:** *Pseudomonas*, Methylotrophy, Autotrophy, Metabolism, Methanol dehydrogenase, Alcohol dehydrogenase

## Abstract

**Background:**

Very few true *Pseudomonas* methylotrophic strains have been described, and in none of them have the pathways for one-carbon (C_1_) substrate metabolism been elucidated.

**Methods:**

The genomes of three *Pseudomonas* strains able to grow on methanol as the sole source of carbon (C) and energy (E) were sequenced and analyzed, and one of the strains was further characterized at the proteomic and physiological level.

**Results:**

None of the three strains possesses a classic methanol dehydrogenase enzyme, and they apparently employ generalist type-I alcohol dehydrogenases (ADHs) to catabolize methanol to formaldehyde. In two of the strains’ genomes, the only complete route encoded for incorporating methylotrophic carbon is the Calvin-Benson-Bassham (CBB) cycle, while other more typical pathways for C_1_-carbon assimilation (serine cycle, ribulose monophosphate cycle) appear incomplete. The indispensability of the QedA1 alcohol dehydrogenase and of ribulose bisphosphate carboxylase for growth on methanol was demonstrated by insertion mutagenesis of the *qedA1* and *cbbL* genes in one of the strains.

**Discussion:**

To the author’s knowledge, all wild-type methylotrophic *Pseudomonadota* (*i.e.*, “Gram-negative bacteria”) so far described employ a specific dehydrogenase distinctively adapted to using methanol as a substrate (MxaFI, XoxFI, or Mdh2). The methylotrophic *Pseudomonas* strains described here lack MDH and employ generalist ADHs, thus demoting methanol dehydrogenase (MDH) from the position of a critical enzyme for methanol utilization and expanding the range of enzymes (and genes) that enable methylotrophy in nature. The second remarkable result of this work is the discovery of the utilization of the CBB cycle by a *Pseudomonas* strain during methylotrophic growth, an absolute novelty for this very relevant bacterial genus.

## Introduction

*Pseudomonas* is a very large and incessantly expanding bacterial genus within the *Pseudomonadota* phylum and *Gammaproteobacteria* class. The genomic and phenotypic richness of the species within this genus is notorious and ever growing (Girard et al., 2021). However, in the past few decades numerous diverse strains were incorrectly classified as *Pseudomonas*, which were later moved to other genera (even to other classes than Gammaproteobacteria) when more rigorous, molecular-based phylogenetics and taxonomy were implemented (Kersters et al., 1996). Due to this, up to the end of the last century, many methylotrophic *Pseudomonas* strains appeared in the literature. However, practically all of these turned out not to be true *Pseudomonas* and were reclassified as *Alphaproteobacteria Methylobacterium*, or *Mycoplana*, or (*Microcyclus*) *Ancylobacter*, or *Methylobacillus*; or *Betaproteobacteria Burkholderia* or *Alcaligenes*. In 1982, Green & Bousfield (1982) conducted a comprehensive revision of the taxonomy of numerous methylotrophic strains previously assigned to the *Pseudomonas* genus: in the end, only three out of the 178 facultative methylotrophic strains studied could be classified as *Pseudomonas*: strain MOX and two fluorescent strains named 42(2) and 42(3), very similar to each other.

At the beginning of this century, when this author and colleagues isolated and began studying another methylotrophic fluorescent *Pseudomonas* strain (PM2) (Pacheco et al., 2003), Dr. Peter Green of NCIB was kind enough to send us the above-mentioned three strains from his culture collection. At the time, growth of the MOX stock sample on methanol could not be achieved, so only strains 42(2) and 42(3) (meanwhile renamed by Dr. Green as P14 and P15, respectively) had the expected phenotype. However, these two strains in our hands proved to be almost indistinguishable, both phenotypically and biochemically.

Other methylotrophic, apparently *bona fide Pseudomonas* strains were described by the group of Prof. Ann Wood at King’s College, UK (Anesti et al., 2004; Boden et al., 2008; Hung et al., 2011). However, these stocks were lost when Dr. Wood’s laboratory shut down in 2010 (A Wood, 2011, pers. comm.). All other fluorescent *Pseudomonas* strains in this author’s private collection were also tested, but none grew on C_1_ compounds as sole sources of carbon and energy (C & E). In 2022, a *Pseudomonas aeruginosa* strain (AAK/M5) was described that could apparently grow on methane. However, the poor quality of the data and analysis presented in that article leaves this author totally incredulous that AAK/M5 really was a methylotrophic strain (see PubPeer commentary De Marco, 2025).

Methylotrophic *Proteobacteria* (*Pseudomonadota*) have been known to oxidize methanol to formaldehyde by exploiting an alcohol dehydrogenase generically called methanol dehydrogenase (MDH), belonging to one of three alternative and homologous, though different, pyrroloquinoline quinone (PQQ)-linked forms of this enzyme which have been exhaustively characterized: calcium-dependent MxaFI (Anthony, 2004), Mdh2 (Kalyuzhnaya et al., 2008) and lanthanides-dependent XoxF (Chu & Lidstrom, 2016). All these isoenzymes are type-I alcohol dehydrogenases and share some degree of sequence similarity among them and with other PQQ-linked alcohol dehydrogenases (ExaA, QedA, BOH, GDH), but they have higher affinities for methanol than for longer or more complex alcohols.

In methylotrophic *Firmicutes* (now *Bacillota*), the same metabolic task is accomplished by NAD-dependent enzymes (Bystrykh et al., 1993; Krog et al., 2013) while methylotrophic yeasts perform methanol oxidation using a peroxisomal peroxide-forming alcohol/methanol oxidase (Aox/Mox) (Yurimoto, Oku & Sakai, 2011).

In strain PM2, growth on alcohols induced the expression of a ca. 70 kDa polypeptide which was recognized by anti-MxaF antibodies, but no amplification was achieved with *mxaF*-specific PCR primers nor significant signal was obtained with an *mxaF* gene probe (Pacheco et al., 2003). In order to understand how these three strains metabolize methanol, their genomes were sequenced. In this work, it will be shown that none of them possesses a typical ‘Gram-negative’ MDH (MxaFI or Mdh2 or XoxFI) and that *Pseudomonas* strain P14 employs an apparently generic alcohol dehydrogenase (ADH) enzyme to oxidize methanol and, even more surprisingly, assimilates carbon by fixing inorganic CO_2_ through the Calvin-Benson-Bassham (CBB) cycle, which, to the best of this author’s knowledge, makes this the first autotrophic *Pseudomonas* strain known to science.

## Materials & Methods

### Bacterial strains, plasmids and culture conditions

Nine other fluorescent *Pseudomonas* strains available in our collection were tested for growth in the same conditions (MM + methanol): *P. putida*—NCIMB 8248, *P. synxantha*—IFO 3913, *P. synxantha* strain G (Pirttilä et al., 2000), *P. fluorescens* ST (Baggi et al., 1983), *P. putida* KT2440—DSM 6125, *P. azotoformans*—IAM 1603, *P. fluorescens*—DMS 50090, *P. syringae pvar. syringae*—DSM10604, *P. aeruginosa* PAO1—DSM 19880 (Table 1).

### Media

Luria-Bertani (LB) (Sambrook & Russell, 2001) and agarized LB were used for general maintenance of the strains. Minimal Medium (MM) was M9 prepared as described in Sambrook & Russell (2001) supplemented with Na/K phosphate buffer 70 mM pH 7, trace metals (TM) (Tuovinen & Kelly, 1973) and a carbon source: methanol 0.2% (v/v) (49.4 mM) or ethanol 0.2% (v/v) (34.3 mM) or glucose 10 mM (1.8 g/L). When needed, agar was added at 16 g/L. Growth in liquid was carried out at 28 °C with shaking (140–150 rpm).

Molar yields were estimated by growing strain P14 in 50/100 mL MM + methanol in C-limited conditions (0.2% v/v) to early stationary phase, obtaining the biomass by filtering cultures through 0.45 µm cellulose filters, drying the filters for 24 h at 50 °C, weighing dry biomass, and matching the result with the number of moles of methanol consumed.

**Table 1:** Bacterial strains used.

**Strain**	**Medium**	**Source**	**Reference**
*Pseudomonas* sp. str. P14	LB/ M9 + TM	DSM-117837	Green & Bousfield (1982)
*Pseudomonas* sp. str. P15	LB/ M9 + TM	DSM-117838	Green & Bousfield (1982)
*Pseudomonas* sp. str. PM2	LB/ M9 + TM	DSM-117839	Pacheco et al. (2003)
*E. coli* str. DH5a	LB	DSM-6897	
*E. coli* str. S17-1	LB/Trim		Simon, Priefer & Pühler (1983)
Plasmid pGEM	Amp		Amplicon cloning vector contained in the pGEM^®^-T-Easy Vector System (Promega™)
Plasmid pK18mob2	Km		Kirchner & Tauch (2003)

**Notes.**

P14, P15 = 42(2) & 42(3) strains.

Trim Trimethoprim 100 mg/L Amp ampicillin 100 mg/L Km kanamycin 50 mg/L

### DNA extraction, genome sequencing and analysis

The three strains (P14, P15 and PM2) were grown in LB medium overnight and biomass was recovered by centrifugation. DNA was obtained by extraction and purification using a Qiaamp^®^ DNA Mini kit. The three genomes were sequenced by STABVida (Caparica, Almada, Portugal): genomic libraries were obtained using a Kapa HyperPrep kit (Roche) and run on an Illumina Platform, paired-end read (2 ×150 bp) sequencer. The three sets of paired reads were quality checked (QUAST) and assembled by Shovill/Megahit (Seemann, 2017) on Galaxy Europe (https://usegalaxy.eu/) (Afgan et al., 2022) and contigs <200 bp were excluded. Type-strains with genome sequences closest to our three strains were discovered through the Type (Strain) Genome Server- TYGS (Meier-Kolthoff & Göker, 2019) available at DSMZ (https://tygs.dsmz.de). Average Nucleotide Identity (ANI) and Average Amino Acid Identity (AAI) levels were computed using FastANI available on Galaxy Europe (Jain et al., 2018). Genome completeness was assessed by BUSCO (Simão et al., 2015). The assemblies’ quality was evaluated using GQC (https://genomeqc.maizegdb.org/) (Manchanda et al., 2020). The assembled genomes were annotated by Mage MicroScope (Vallenet et al., 2019) and the results were submitted to the European Nucleotide Archive (https://www.ebi.ac.uk/ena/browser) (BioProject number PRJEB89234, Accession numbers GCA_965286985 (PM2), GCA_965286995 (P14), and GCA_965287005 (P15)).

Homologues of QedA1 or CbbL were found by BLASTp searches (Altschul et al., 1990). The sequences were aligned using MUSCLE (Edgar, 2004), the alignments cured using GBlocks (Talavera & Castresana, 2007) and phylogenetic trees were inferred by PhyML (Guindon et al., 2010) as made available on the Phylogeny.fr platform (Dereeper et al., 2008).

### Protein extraction and proteomic analysis

Strain P14 was grown in MM supplemented with methanol 0.2% or ethanol 0.2% or glucose 10 mM and biomass was collected in mid-exponential phase (OD_600_ = 0.25, 0.7, and 1.2, respectively) by centrifugation at 4 °C. Pellets were washed once in cold PBS buffer (potassium phosphate 22 mM, NaCl 100 mM, pH 7.0), then resuspended in 0.5 mL of the same and sonicated on ice with a SONICS^®^ Ultrasonic Processor VCX130 fitted with a 3 mm probe, during four cycles of 30 s on/30s off, duty cycle 50% (2 s on/off), 130 W, 20 kHz. The lysates were centrifuged at 16,000×g 4 °C for 20 min and the supernatants were carefully separated from the pellets and frozen in small aliquots at −80 °C until used.

Extracts were sent to i3S (University of Porto, Porto, Portugal) Proteomics Scientific Platform to be analyzed by mass spectrometry-based proteomics on a Vanquish neo LC coupled to an Orbitrap Eclipse MS (Thermo Fisher Scientific). Protein extracts were digested with trypsin. Protein identification and quantification were performed using the Proteome Discoverer software following the LFQ—Label Free Quantification approach using strain P14 annotated genome as reference. Automatic KO assignment and KEGG mapping of the active proteome in each condition was performed using BlastKOALA (Kanehisa, Sato & Morishima, 2016) at keg.jp.

The alcohol dehydrogenase activity in cell-free extracts was measured spectrophotometrically essentially as described in Day & Anthony (1990). Mean values and standard deviation were calculated from three independent samples measured five times each.

### Production of mutants

Mutants of strain P14 with inactivated *qedA1* or *cbbL* genes were produced by mutagenic plasmid insertion (MPI) technique (details in the Supplementary Material). A 200-bp stretch of the *qedA1* gene was obtained by PCR amplification using a mutagenic primer pair (mQf + mQr): each primer had a mismatched base that introduced in-frame stop codons in the sequence of the amplified gene segment (see Table 2 and Fig. S2). The mutated gene segment was ligated to the transferable narrow-host-range suicide vector pK18mob2 (Kirchner & Tauch, 2003) and the resulting two constructs (pK18-mQ1 and pK18-mQ2, with the insert in either orientation) were checked by sequencing and introduced into *E. coli* str. S17-1 by electroporation. The transformed clones of S17-1 were then used in filter conjugation with *Pseudomonas* strain P14 as a recipient. Transconjugants were selected in Cetrimide agar (OXOID^®^) + kanamycin. Two kanamycin-resistant transconjugant P14 clones of each conjugation were selected and PCR checked with primers mQf, mQr, PUCF or PUCR (according to insert orientation) for genomic integration: all yielded the expected amplification patterns and one of each orientation (P14-mQ1A and P14-mQ2C) was further checked with primer pairs KmF+KmR, mQF+mQR or UpQ+DpQ (no amplification expected in this latter case), using wild-type strain P14 as control. Some amplicons were checked by Sanger sequencing to confirm the presence of the planned nonsense mutations. The two transconjugants were kept for further analysis.

**Table 2:** PCR primers used.

**Name**	**Description**	**Sequence (5′–3′)**
mQf	Mutagenic fwd primer for gene *qedA1*	ATGACAATATGATCGCTACCCGCC
mQr	Non-mutagenic rev primer for gene *qedA1*	GTGCCCATGCCCTACTGCAG
PUCF	Primer pairing with plasmid pK18mob2 downstream of the MCS	AGGGTTTTCCCAGTCACGAC
PUCR	Primer pairing with plasmid pK18mob2 upstream of the MCS	ACACAGGAAACAGCTATGAC
mCf	Mutagenic fwd primer for gene *cbbL*	ATGGCTAAGACCTAGAAGGC
mCr	Mutagenic rev primer for gene *cbbL*	CCTAGAACAGGTCGATGGG
UpQ	Insertion control fwd primer for *qedA1*	TTCAGGAGGCCAACATGGAG
DpQ	Insertion control rev primer for *qedA1*	GCTCCTACGACGGGCTGT
UpC	Insertion control fwd primer for *cbbL*	TGGTTGGTGCCGATAAGA
DpC	Insertion control rev primer for *cbbL*	AAGCGGACGTCTTCAAG
SAf	Non-mutagenic fwd primer for a “conserved gene of unknown function”	GCATTCATTCGGTCACGGTAAG
SAr	Non-mutagenic rev primer for a “conserved gene of unknown function”	GCGATCGACTGTATCGTCCTG
KmF	Fwd primer for pK18mob2 Km^R^ gene	GGCGATAGCTAGACTGGG
KmR	Rev primer for pK18mob2 Km^R^ gene	CGATTCCGAAGCCCAACC
Mxa f1003	Fwd primer for *mxaF* gene (McDonald & Murrell, 1997)	GCGGCACCAACTGGGGCTGGT
Mxa r1561	Rev primer for *mxaF* gene (McDonald & Murrell, 1997)	GGGCAGCATGAAGGGCTCC
PQQDH- 215F	Fwd primer for *mdh2* gene (Kalyuzhnaya et al., 2008)	CAGCGCTACAGCCCGCTCAAG
PQQDH-1805R	Rev primer for *mdh2* gene (Kalyuzhnaya et al., 2008)	GTACTGCTC GCCGTCCTGCTCCC

**Notes.**

fwd/rev forward / reverse MCS multi-cloning site within *lacZ*′

A similar insertion-mutagenesis scheme was applied to the gene predicted to encode RuBisCO large subunit (*cbbL*) using mutagenic primers mCf + mCr (see Table 2 and Fig. S4). Two kanamycin-resistant transconjugant P14 clones of each type were PCR checked with primer pair mCf+PUCF (or PUCR) for genomic integration in the expected *locus*: all were positive and one of each orientation (P14-mC2 and P14-mC66) was further checked through specific PCR reactions and Sanger sequencing and was kept for further analysis.

A parallel negative-control non-mutagenic plasmid-insertion strategy was designed for insertion into a “conserved gene of unknown function” (ORF METHP14 _60077 in the published genome sequence, see diagram of Fig. S5). This approach integrated the pK18mob2 vector into a plausibly neutral *locus* of the genome in a way expected not to affect strain P14’s methylotrophic traits. The transconjugant strain obtained was named P14-SA1 and plasmid integration in the expected site was confirmed by PCR and amplicon sequencing.

## Results

Strains P14 and P15 are fluorescent *Pseudomonas* that grow on methanol or sodium formate as sole C & E sources consistently but slowly (doubling times estimated ca. 33 h/50 h) and with comparatively low yields (5 ± 1.3 g/mol methanol). Growth on C_1_ substrates could only be achieved in flasks with vigorous lateral shaking (orbital shaking was insufficient) while growth in 96-well microtiter plates was not attained with methanol or formate, just with glucose or complex media. Similar results had been obtained with strain PM2 (Pacheco et al., 2003). All three strains grew well and fast on complex media or MM + glucose, suggesting that methylotrophic growth is a less favorable type of metabolism for them. Recently strain PM2 from our stock culture lost the ability to grow on methanol (or ethanol), so this strain could no longer be used in physiological tests in this work.

In order to ascertain what type of MDH strains P14 and P15 employed, PCR amplification of genes *mxaF* and *mdh2* was attempted using specific primers (Kalyuzhnaya et al., 2008; McDonald & Murrell, 1997), but repeated efforts were unproductive.

Genomic DNA samples were then sequenced and the results analyzed as described in the Methods section. Sequencing statistics are summarized in Table 3. The genome of strain PM2 was assembled into 700 scaffolds (of which 372 ≥ 200 bp) for an overall sum sequence of 6.5 Mbp containing 6,558 genomic objects (of which 6,419 coding ORFs). BUSCO analysis score for genome completeness was 99.9%. In the case of strain P14, 464 scaffolds were obtained (315 ≥ 200 bp long) yielding a global genome of 6.1 Mbp with 5,999 genomic objects (5,865 coding) and a 99.6% completeness level. Strain P15′s genome was 99.96% identical to P14′s, so the bulk of the subsequent work was focused just on strain P14.

**Table 3:** Genome sequencing and assembly results (Megahit 200 bp-limited).

**Strain**	**P14**	**P15**	**PM2**
Number of reads	9,365,400	9,501,776	10,693,706
Number of scaffolds	315	310	372
Total size of scaffolds (bp)	6,121,800	6,109,190	6,525,500
Useful amount of scaffold sequences (≥25K nt) (bp)	5,092,893	5,154,570	4,758,122
% of estimated genome that is useful	84.88155	85.9095	79.30
Longest scaffold (bp)	387,841	223,114	140,005
Shortest scaffold(bp)	200	200	200
Number of scaffolds >1K nt	190	190	250
Number of scaffolds >10K nt	126	127	173
Number of scaffolds >100K nt	10	8	7
N50 (bp)	57,600	58,760	39,365
L50	27	31	48
%A	19.088	19.046	20.023
%C	30.943	30.950	29.853
%G	30.928	30.939	29.982
%T	19.041	19.066	20.142
%GC	61.871	61.889	59.835
Total Number of Ns	0	0	0
%N	0	0	0
Median sequence size (bp)	2,830	3,445	8,423
Mean sequence size (bp)	19,434.3	19,707.1	17,541.7

DSMZ’s TYGS server genome-based phylogenetic analyses indicated that strain PM2 is most similar to *P. salmasensis* SWRI126 (calculated digital DNA-DNA hybridization value d_4_ = 65.7%) and *P. lactis* DSM-29167 (d_4_ = 64.6%). On the other hand, strains P14 and P15 scored most similar to *P. putida* NBRC 14164 (d_4_ = 40.2%) and *P. kurunegalensis* RW1P2 (d_4_ = 39.3%).

**Figure 1:**
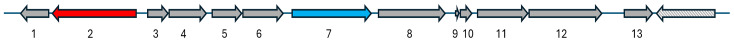
Organization of the locus containing gene *qedA1* in strain P14 (identical in strain P15). 1. Pentapeptide repeat family protein (or *qedB*) 2. Quino(hemo)protein alcohol dehydrogenase *qedA1* 3. Cytochrome c550, associated with quino(hemo)protein alcohol dehydrogenase *qedC* or *exaB* 4. Extracellular substrate-binding protein associated with quino(hemo)protein alcohol dehydrogenase 5. Uncharacterized protein PP2677 / sulfur-oxidizing protein SoxY 6. MBL-fold metallo-hydrolase superfamily/cyclase 7. Quino(hemo)protein alcohol dehydrogenase, PQQ-dependent, *qedA2* 8. Aldehyde dehydrogenase *aldB* 9. Coenzyme PQQ precursor peptide A (*pqqA*) 10. Coenzyme PQQ synthesis protein D (*pqqD*) 11. Alcohol dehydrogenase (ORF_50082) 12. Sensory box histidine kinase/response regulator 13. Hypothetical protein.

Both PM2 and P14/P15 genomes contained genes for the catabolism of methylamine (*mauAB*), formaldehyde (*fdhA*, *mdo*/*fdm*) and formate (*FrmB*/*Fdh1*), but no gene was annotated as methanol dehydrogenase. This result was manually confirmed by BLASTp searches using MxaF or Mdh2 or XoxF as queries. In all cases, only distant hits were retrieved (all of which had been automatically annotated as “alcohol dehydrogenases – ADH”), with low/medium similarity values (55–79%). Indeed, several ADHs were annotated on these genomes: *adhAB* E.C.:1.1.5.5; *ahr*/*yahK* E.C.:1.1.1.2; *adhP*/*yiaY*/*frmA*/*adhE* E.C.:1.1.1.1; *exaA*/*qedA*/*pedE* E.C.:1.1.2.8 (this last only in strains P14/P15). In both these two strains, two *qedA* genes were annotated in close proximity: one (called *qedA1* in this paper) in a small operon with a second gene (*qedB*, encoding a pentapeptide-repeat family protein) (Promden et al., 2008), plus another called *qedA2* in a larger adjacent operon containing a copy of *exaB* (cytochrome c550), an aldehyde dehydrogenase B gene (*aldB*), *pqqA* and *pqqD* (genes involved in the synthesis of PQQ) and one more alcohol dehydrogenase (ORF_50082) (Fig. 1).

**Figure 2:**
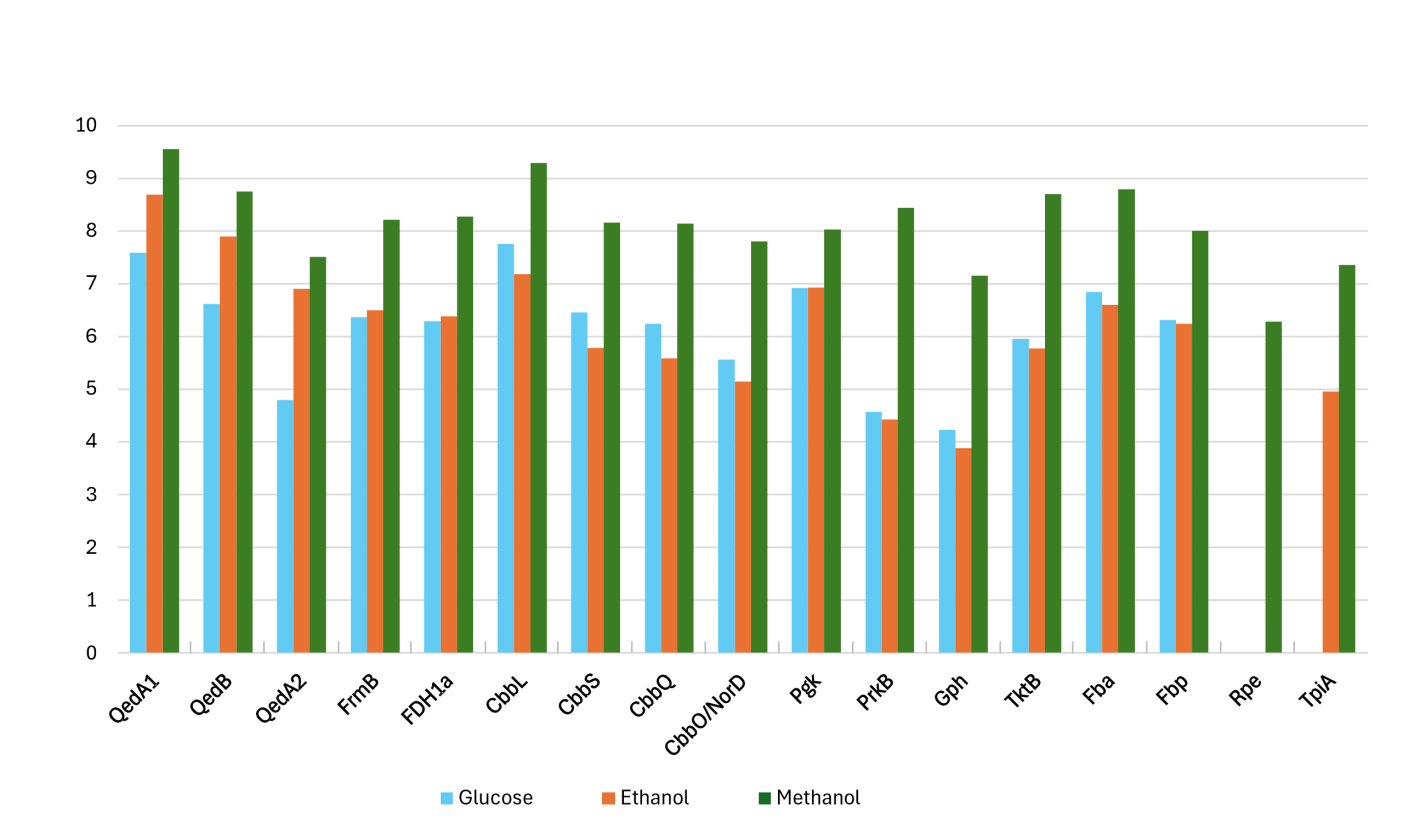
Log-transformed normalized abundances (from proteomic analysis) of strain P14’s proteins involved in the utilization of methanol (growth substrate methanol or ethanol or glucose). QedA1, QedB, QedA2: alcohol dehydrogenases. FrmB, FDH1a = formate dehydrogenases. CcbL, CcbS, CcbQ, CcbO/NorD = ribulose bis-phosphate carboxylase; Pgk, phosphoglycerate kinase; PrkB, phosphoribulokinase; Gph, phosphoglycolate phosphatase; TktB, transketolase; Fba, Fructose-bisphosphate aldolase; Fbp, Fructose 1,6 phosphatase; Rpe, Ribulose-phosphate epimerase; TpiA, triose-phosphate isomerase.

Proteomic data from strain P14 showed that QedA1 was the most abundant polypeptide in methanol-grown cells, while it was much less abundant (ca. 7-fold and 92-fold, respectively) in ethanol- or glucose-grown cells. QedB was also expressed much more with methanol than with ethanol or glucose (Fig. 2 and Fig. S1). Other alcohol dehydrogenases were much less present in methanol-grown cells (*e.g.*, QedA2 ca. 100 times less, ORF_50082 ca. 1,000 times less). Also, mutant strains P14-mQ1A and P14-mQ2C, in which gene *qedA1* was interrupted (see Fig. S2), were unable to grow on methanol (Supplemental Information 9). Considering the way deactivation of *qedA1* was performed, it is very likely that, in these mutants, gene *qedB* is also silenced due to uncoupling from its natural promoter. However, it was previously demonstrated that interrupting *qedB* in *P. putida* H5 did not abolish alcohol dehydrogenase (ADH-I) activity (Promden et al., 2008), so it is unlikely that a lack of QedB should cause a lack of alcohol dehydrogenase in strain P14. All these data support the contention that QedA1 is the enzyme responsible for the oxidation of methanol in strain P14.

QedA1 and QedA2 were compared to 38 other type-I alcohol dehydrogenases of the various groups (and six type-II ADHs as an outgroup). After aligning, QedA1 showed high similarity to generalist alcohol/ethanol dehydrogenases and less to methanol dehydrogenases of the various types (MxaF, XoxF, Mdh2), showing alignment blocks and gaps conserved with generalist ADHs throughout the alignment (Fig. S3). QedA1 was almost identical (99.8%) to quinoprotein ethanol dehydrogenase of *P. putida* KT2440 (WP_010953593.1) and very high identity and similarity values were found with other enzymes annotated as ethanol/ or alcohol/ or ethanol/methanol dehydrogenases from *Pseudomonas* strains of the *putida* group. However, none of these strains is known to be methylotrophic. QedA2 was most similar to PQQ-dependent alcohol dehydrogenase PedH of *Pseudomonas* (multispecies, WP_021784002.1) and quinoprotein ethanol dehydrogenase precursor ExaF of *Methylobacterium extorquens* AM1 (ACS39011.1).

**Figure 3:**
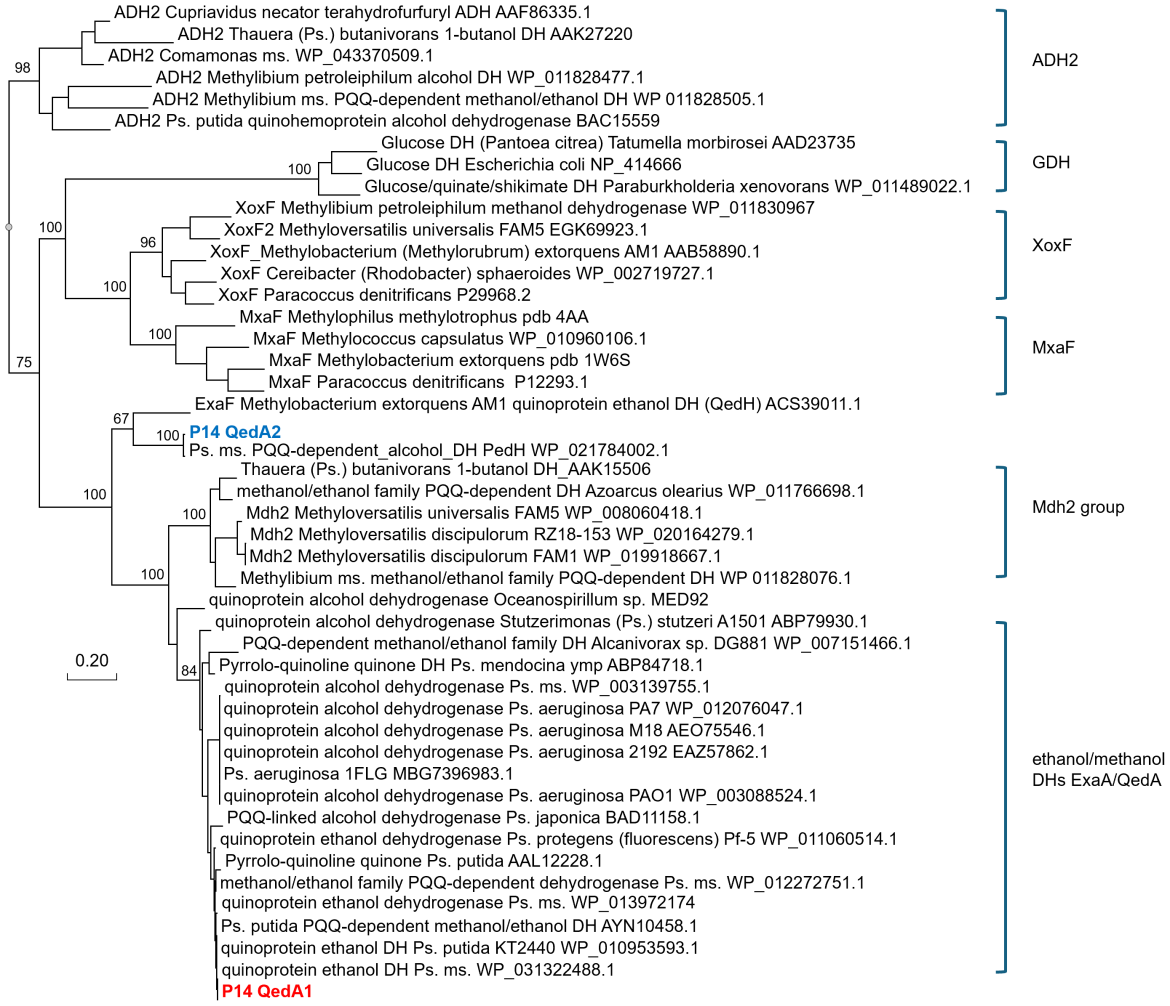
Phylogenetic tree of alcohol dehydrogenases. Ps., Pseudomonas ms., multispecies DH(s), dehydrogenase(s) Values at the nodes are bootstrap support values over 100 replicates.

A phylogenetic tree was produced from this alignment (Fig. 3) which corroborates the same idea: strain P14′s QedA1 falls unquestionably within a group composed of ethanol/methanol dehydrogenases most of which belong to *Pseudomonas* species with 84% bootstrap support, while each MDH (MxaF, XoxF, Mdh2) belongs in its own undisputed branch (with bootstrap support between 96% and 100%). QedA2 grouped with PedH and ExaF.

Alcohol dehydrogenase assays of methanol-grown strain P14 cell-free extracts showed that both methanol and ethanol can be oxidized by the enzymes induced in these conditions and that the specific activity measured was consistently and significantly higher with ethanol (83.60 ± 28.01 µmol mg prot^−1^ min^−1^) than with methanol (67.65 ± 32.12 µmol mg prot^−1^ min^−1^). Such activity is probably mostly ascribable to QedA1, by far the highest induced ADH in these growth conditions (>100 times higher than QedA2). This observation suggests that, despite not being specifically evolved to catabolize methanol, QedA1 can nevertheless be efficiently employed with this substrate, provided the host’s cell regulatory network adequately recognizes methanol as an inducer.

P14′s QedA1 is very similar in sequence (95%) to ethanol dehydrogenase QedA of *P. putida* (A8R3S4 ⋅ QEDH_PSEPU). The two secondary structures deduced from the two amino acid sequences using PROTEUS2 (http://www.proteus2.ca/proteus2/) (Montgomerie et al., 2008) showed that these two proteins are almost identical. The only difference is an eight amino acid-longer N-terminal of the mature peptide of P14′s QedA1. Despite these striking similarity levels, Toyama et al. (1995) reported no activity for *P. putida* QedA with methanol as substrate.

However, other type-I PQQ-dependent ethanol dehydrogenases from *Pseudomonas* have been observed to oxidize methanol too (Görisch & Rupp, 1989): indeed, several of them are explicitly defined as “ethanol/methanol dehydrogenases”. In a similar way, mutant strain Tp9002 of *Paracoccus* (*Thiosphaera*) *pantotrophus* was found to acquire methanol-degrading capacity by de- and up-regulating the expression of its “dye-linked ethanol dehydrogenase” (Ras et al., 1995).

Taking into consideration all these findings, it becomes difficult to recognize a clear frontier between MDHs and ADHs.

As for the conversion of formaldehyde to formate, three alternative pathways were available as per the genome annotation: a glutathione-independent formaldehyde dehydrogenase (FdhA, EC: 1.2.1.46); a formaldehyde dismutase (Fdm, EC:1.2.98.1); or a two-reaction chain of S-(hydroxymethyl)glutathione dehydrogenase (FrmA, EC:1.1.1.284) + S-formylglutathione hydrolase (FrmB, EC:3.1.2.12). The proteome analysis showed that all three were expressed when P14 was grown on methanol, although FdhA at much lower levels than the latter two. For the final oxidation of formate to CO_2_, a formate dehydrogenase activity (EC:1.17.1.9) was predicted from the genome sequence: indeed, gene *fdh1A* was highly expressed on methanol, much less (532 times) on ethanol and not at all on glucose.

As for carbon incorporation into biomass, both the serine and RuMP pathways were incompletely annotated in both strains P14/P15 and PM2. However, on strains P14/P15′s genomes, a practically complete CBB cycle was predicted. No gene was explicitly scored as sedoheptulose-1,7-bisphosphatase by the automatic annotation, but this is a situation common to other autotrophic bacteria whereby this enzymatic activity (EC:3.1.3.37) is supplied by a bifunctional fructose-1,6/sedoheptulose-1,7-bisphosphatase (EC:3.1.3.11) (Jiang, Wang & Wen, 2012; Sporre et al., 2023; Yoo & Bowien, 1995). Indeed, fructose-1,6- bisphosphatase Fbp and the other enzymes associated with the CBB cycle, including the polypeptides encoded by the *cbb* operon, were all very highly expressed when strain P14 grew on methanol. Curiously, some of these enzymes were also somewhat expressed when the source of carbon was glucose while their lowest levels of expression were seen on ethanol (Fig. 2 and Fig. S1).

Mutants of strain P14 in which gene *cbbL* was inactivated by insertion and nonsense mutations (mC2 and mC66) indeed were unable to grow on methanol (see Fig. S4 and Supplemental Information 9). This, together with the very high expression levels of *cbb* genes measured during methanol utilization, corroborates the hypothesis that strain P14 incorporates C_1_ carbon at the level of CO_2_ through the CBB cycle.

A BLASTp search showed that two more strains within the *Pseudomonas* genus carry highly identical *ccbL* in their genomes: *P. asiatica* strain P1 (CP084714.1) (Li et al., 2023) and *P. monteilii* str. CY06 (GCF_002835905.1) (Ma, Cai & He, 2019).

Aside from these two *Pseudomonas* hits, BLASTp searches with CbbL from strain P14 retrieved less similar results (identity between 90% and 86%) with several other *Gammaproteobacteria* species (*Ectothiorhodospira*, *Thermomonas*, *Nevskia*, *Thioalkalivibrio*, *Nitrosococcus*, *Enterovibrio*, *Grimontia*, *Methylophaga*, *etc.*), but also *Betaproteobacteria* (*Tepidimonas*, *Thiomonas*, *Methyloversatilis discipulorum*) and *Alphaproteobacteria* (*Sinisalibacter*).

A 30 kbp region around the *cbbL* gene was found to be identical between str. P14 and *P. asiatica* P1 and nearly identical (just 1 mismatch) with *P. monteilii* CY06. In strain P14, this genomic region is comprised between two *loci* containing transposase elements and a resolvase gene, which hints at a transposition event as the origin of this genome segment in the common ancestor of these three *Pseudomonas* species. It also contains a large collection of other genes involved in the CBB cycle (the RuBisCO operon transcriptional regulator RbcR, triose-phosphate isomerase, ribulose-phosphate 3-epimerase, phosphoglycerate kinase, fructose-1,6-bisphosphatase, phosphoribulokinase, fructose-bisphosphate aldolase, transketolase, CbbL, CbbS, CbbQ, CbbO) and a formate dehydrogenase gene.

However, the Average Nucleotide Identity (ANI) levels between strain P14′s genome and *P. monteilii* CY06 (95.76%) or *P. asiatica* P1 (89.9%) were below the suggested intra-species threshold of 99.5% (Rodriguez-R et al., 2024). Also, such disparate geographic and ecological origins (UK garden soil, Chinese shrimp aquaculture pond, and Chinese landfill drainage channel) don’t support the hypothesis of a recent common origin of the three strains. So, it seems reasonable to imagine that in the future strains P14 and P15 will be found to belong to a novel *Pseudomonas* species.

Very recently (May 2025), the genome sequences of six new strains isolated from seawater (NPDC team, 2025, pers. comm.) belonging to a different species, *P. hunanensis*, have been made available in GenBank and through the Natural Products Discovery Center’s website (https://npdc.rc.ufl.edu). Three of these strains (NPDC-76887, NPDC-76888, and NPDC-76889) also possess the 30 kbp genomic region, containing gene *cbbL* and the other genes involved in the CBB cycle, identical or nearly identical (one bp mismatch) to strain P14. These three strains of *P. hunanensis* showed 95.4% ANI to strain P14. Nothing is known about the metabolic properties of these isolates.

In all five abovementioned *Pseudomonas* strains, the complement of enzymes predicted from the genome sequence is exactly the same as in P14, with a complete CBB cycle, so it is sensible to predict the potential for autotrophic growth for all of them.

The uncertainty persists about the mode of methanol-C incorporation by strain PM2 since, beyond the serine and ribulose monophosphate cycles, also the CBB cycle was incomplete, judging from the genome annotation (no *cbbLS* nor phosphoribulokinase). And since this strain has meanwhile lost its ability to grow on methanol, it is impossible to put hypotheses to the test.

## Discussion

This study elucidates for the first time in detail the utilization of methanol by a genuine strain of the *Pseudomonas* genus. All the data collected prove that strain P14 utilizes an unusual enzyme to oxidize methanol and, even more unexpectedly, fixes C_1_ carbon at the level of CO_2_ using the Calvin-Benson-Bassham (CBB) cycle. This means that this *Pseudomonas* strain has evolved to express a competent methanol dehydrogenase activity employing a generical alcohol/ethanol dehydrogenase gene while also developing a unique way to incorporate the resulting carbon flux.

Most methylotrophic *Proteobacteria* (*Pseudomonadota*) incorporate C_1_ carbon at the level of formaldehyde or formate, through the serine pathway or the ribulose monophosphate cycle. But these pathways are both incomplete in the genome of strain P14 while the CBB cycle appears complete and highly active in methanol-grown cells. This makes strain P14 the first ever described autotrophic *Pseudomonas* strain.

Three more *Pseudomonas* species, the genomes of which have recently been sequenced and published, also encode a complete CBB cycle, but the absence of further data prevents us from concluding whether they can indeed fix CO_2_ or whether they are capable of methylotrophic growth.

Strain P14 grows consistently on methanol as its sole source of C & E, although on this substrate its doubling times are long and its molar yields are low. Additionally, the dehydrogenase activity of cell-free extracts appears lower using methanol as substrate than with ethanol. Strain PM2 had also shown low efficiency when growing on methanol (Pacheco et al., 2003). All these facts suggest that these *Pseudomonas* strains may have (evolutionarily) recently adapted to the methylotrophic lifestyle by tweaking the expression of their alcohol dehydrogenases. It is apparently common to encounter fluorescent *Pseudomonas* strains on plant leaves or within plant tissues (Ou et al., 2019; Pirttilä et al., 2000) where methanol (once upon a time called “wood spirit”) is relatively abundant (Dorokhov, Sheshukova & Komarova, 2018). However, most true *Pseudomonas* thus far probed were not able to grow solely at the expense of C_1_ compounds. It is though possible that *in planta* these bacteria lead a mixotrophic existence, *i.e.,* using methanol just as an additional source of energy, while incorporating carbon from more complex substrates. Conceivably, the three strains described in this work took a further evolutionary leap leading to self-sufficient methylotrophy by acquiring the ability to assimilate C_1_ carbon.

*Pseudomonas* is a very diverse and large genus, but methylotrophic strains truly belonging to this clade are few. This novel information on strain P14 considerably enriches the description of the genus further widening its already extensive versatility.

## Conclusions

In this paper, a methylotrophic strain of fluorescent *Pseudomonas*, strain P14, is described in detail. The data accumulated prove that strain P14 a) does not possess a classical MDH enzyme, instead employing a generic alcohol/ethanol dehydrogenase to degrade methanol; and b) assimilates the carbon derived from methanol at the level of CO_2_ through the Calvin-Benson-Bassham (CBB) cycle. As such, strain P14 is not only one of very few true *Pseudomonas* methylotrophic examples, but it also shows that a methanol dehydrogenase is not a requirement to grow on methanol. It is also the first strain within this genus to be proven to assimilate inorganic carbon (CO_2_) and grow autotrophically within a genus commonly considered as a canonical paragon for heterotrophic bacteria.

By genome sequence comparison, it is also plausible that other *Pseudomonas* strains, reported by other laboratories, share these traits: it will be interesting to establish this fact in the future.

More in general, these results show that genomic plasticity, probably attained through lateral gene transfer, can go as far as to reshape such a fundamental trait as the mode of carbon assimilation in *Pseudomonas*, accrediting to this genus even more genetic and metabolic malleability than so far acknowledged.

##  Supplemental Information

10.7717/peerj.20614/supp-1 Supplemental Information 1 Plasmid Insertion Mutagenesis scheme for gene qedA1 inactivation Opposite orientation of inserted vector = strain P14-mQ2C. * = nonsense mutation

10.7717/peerj.20614/supp-2 Supplemental Information 2 Plasmid Insertion Mutagenesis scheme for gene cbbL inactivation. Opposite orientation of inserted vector = strain P14-mC66. * = nonsense mutation

10.7717/peerj.20614/supp-3 Supplemental Information 3 Scheme of Plasmid Insertion into ORF_60077 (ORF METHP14_60077)

10.7717/peerj.20614/supp-4 Supplemental Information 4 Log-transformed expression fold changes of strain P14’s proteins involved in the utilization of methanol Growth substrate methanol or ethanol or glucose—the smallest expression level for each substrate was equaled to 1

10.7717/peerj.20614/supp-5 Supplemental Information 5 Section of a multiple alignment (MUSCLE) of ADHI sequences (region 1)

10.7717/peerj.20614/supp-6 Supplemental Information 6 Section of a multiple alignment (MUSCLE) of ADHI sequences (region 2)

10.7717/peerj.20614/supp-7 Supplemental Information 7 Section of a multiple alignment (MUSCLE) of ADHI sequences (region 3)

10.7717/peerj.20614/supp-8 Supplemental Information 8 Section of a multiple alignment (MUSCLE) of ADHI sequences (region 4)

10.7717/peerj.20614/supp-9 Supplemental Information 9 Culture growth and enzyme activity data

10.7717/peerj.20614/supp-10 Supplemental Information 10 Raw data of proteomics results Raw data obtained from the proteomics analysis of methanol- or ethanol- or glucose-grown strain P14.

## Additional Information and Declarations

### Data Availability

The following information was supplied regarding data availability: The genomes are available at the European Nucleotide Archive: PRJEB89234, GCA_965286985 (PM2), GCA_965286995 (P14), and GCA_965287005 (P15).
