# Supplementary figures and images for: Autotrophic methylotrophy with no methanol dehydrogenase (MDH) in a strain of fluorescent Pseudomonas
Source: PeerJ. 2026 Feb 2;14:e20614. doi: 10.7717/peerj.20614 (PMC12875217; doi:10.7717/peerj.20614)

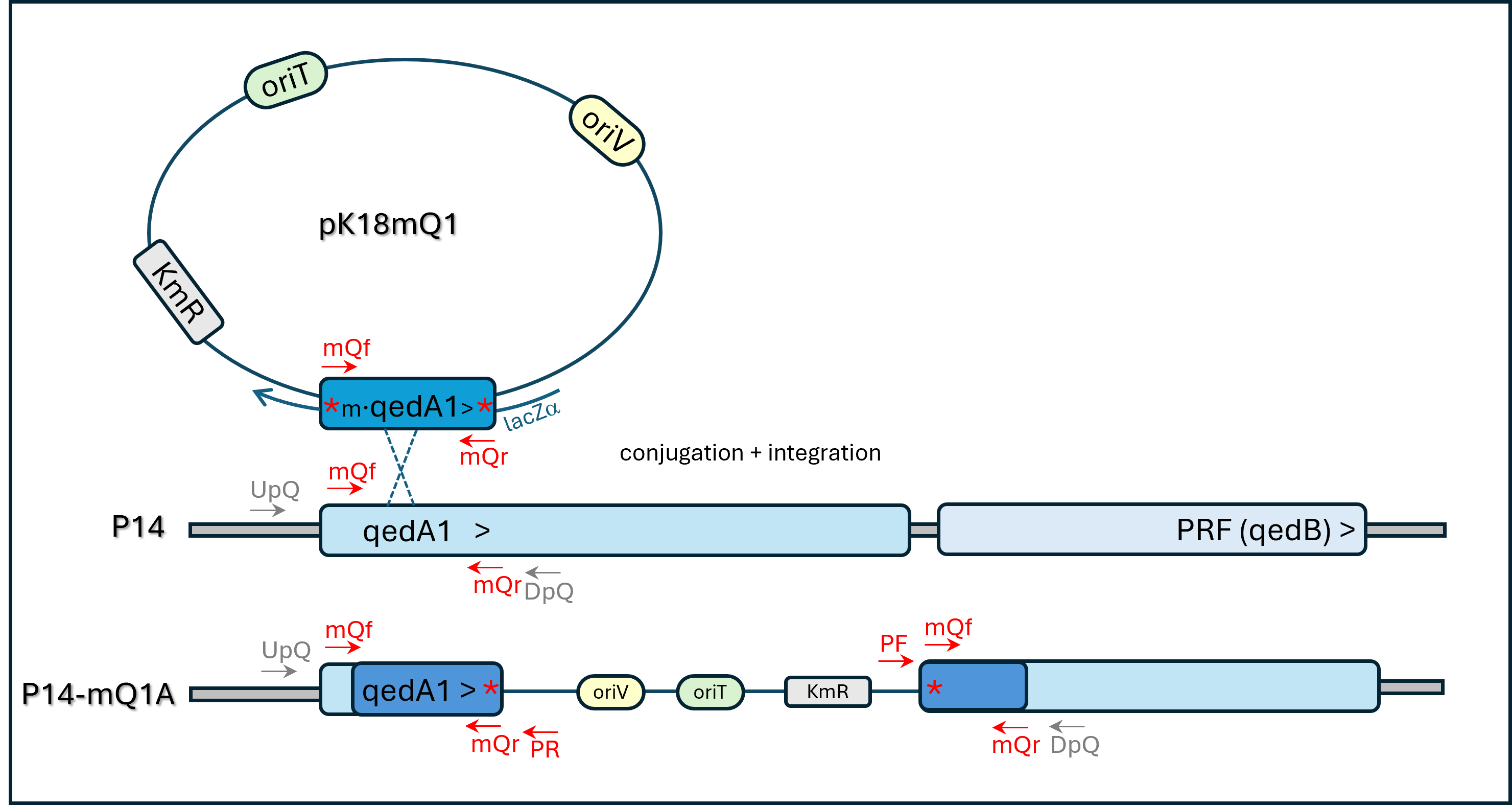

Supplement: Supplemental Information 1 — Opposite orientation of inserted vector = strain P14-mQ2C. * = nonsense mutation [file peerj-14-20614-s001.png]

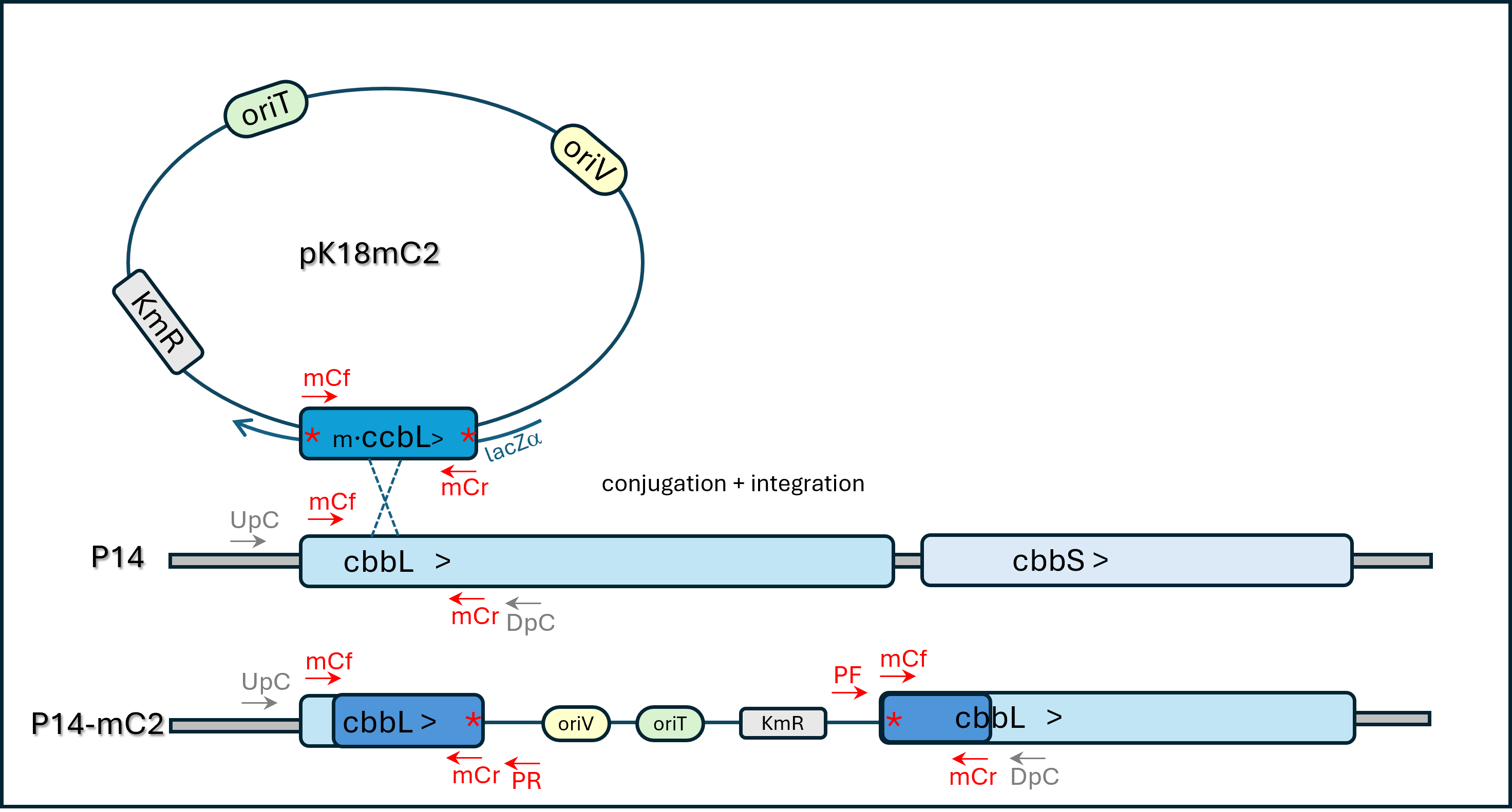

Supplement: Supplemental Information 2 [file peerj-14-20614-s002.png]

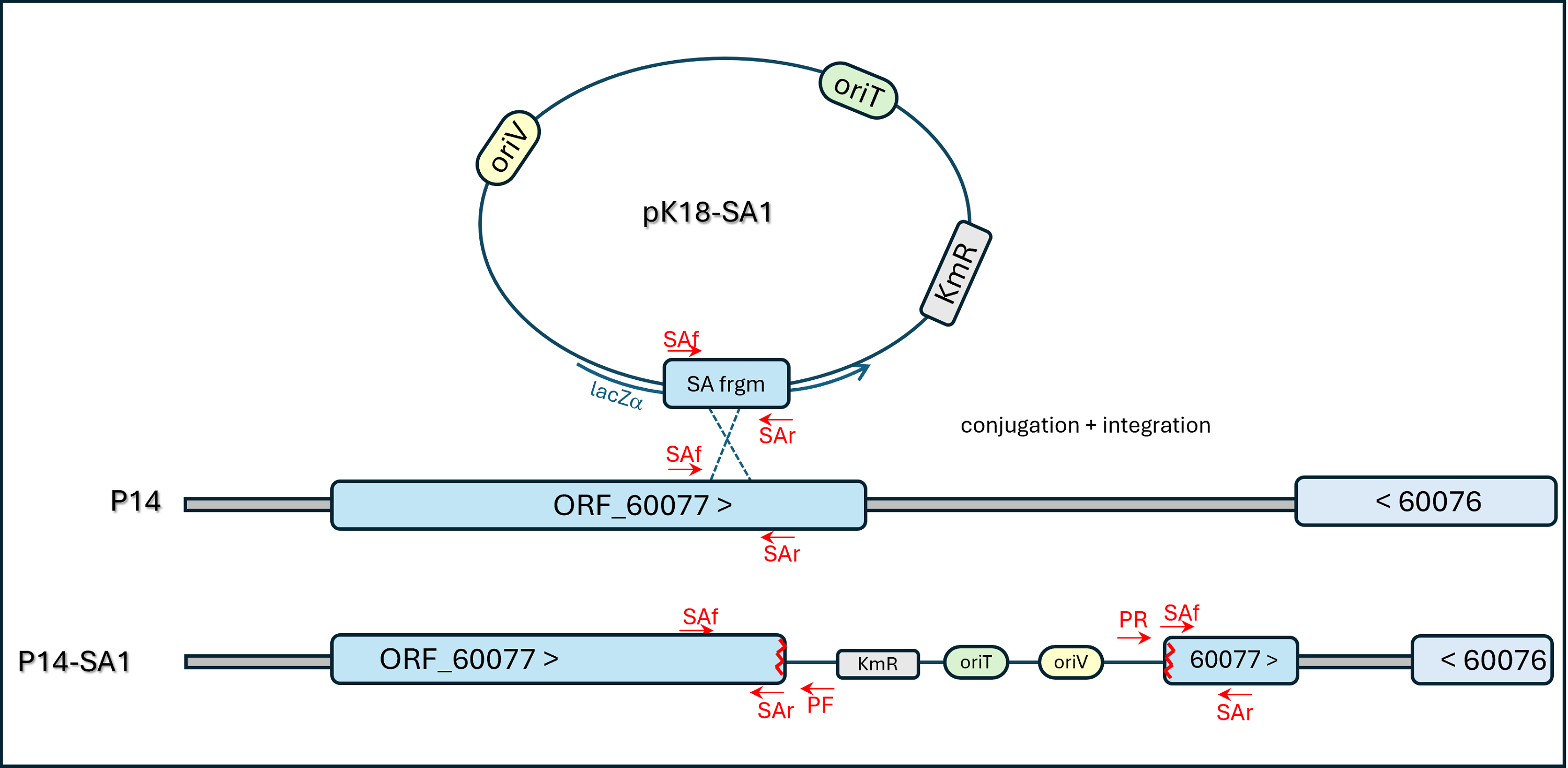

Supplement: Supplemental Information 3 [file peerj-14-20614-s003.png]

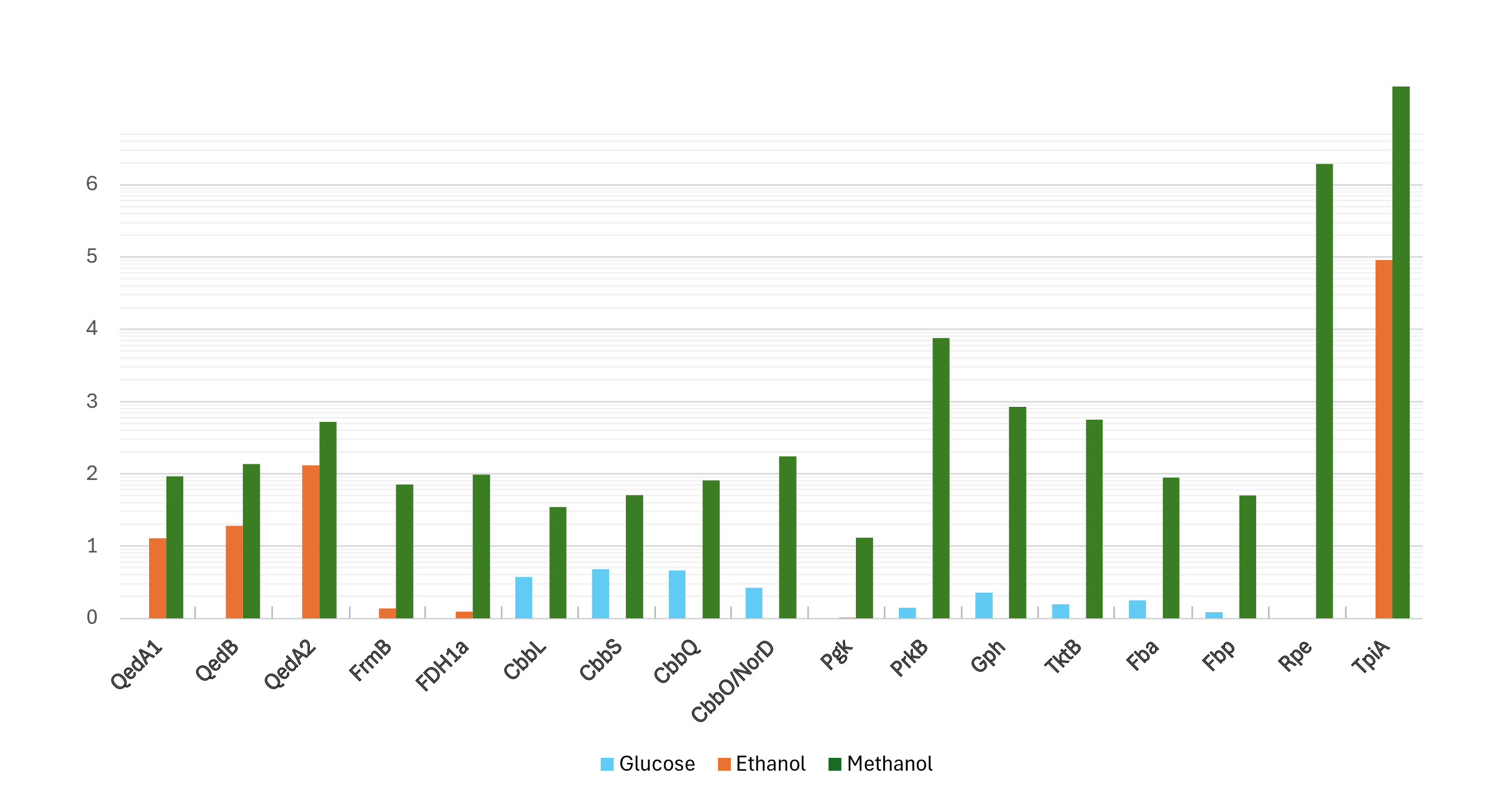

Supplement: Supplemental Information 4 — Growth substrate methanol or ethanol or glucose—the smallest expression level for each substrate was equaled to 1 [file peerj-14-20614-s004.png]

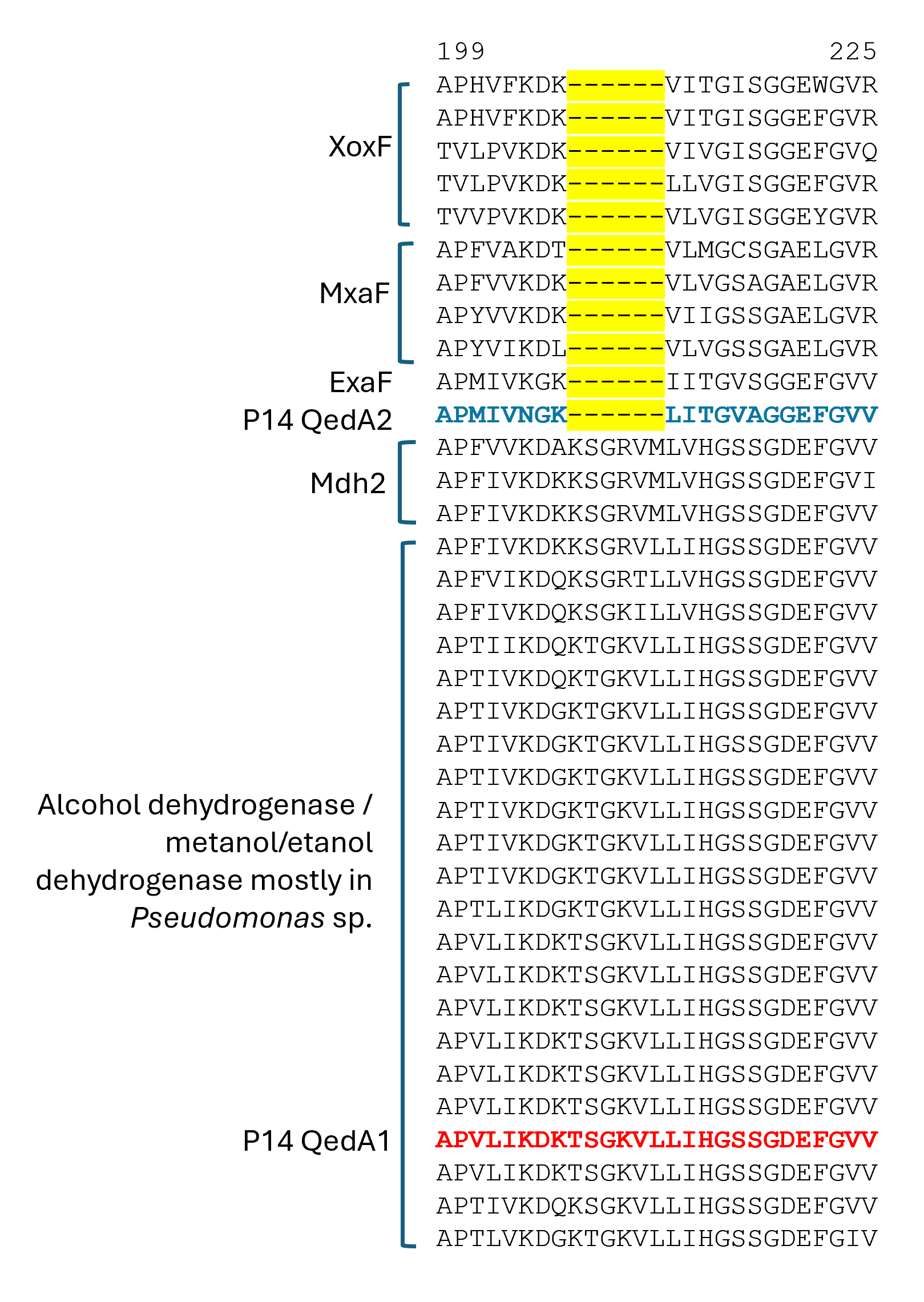

Supplement: Supplemental Information 5 [file peerj-14-20614-s005.png]

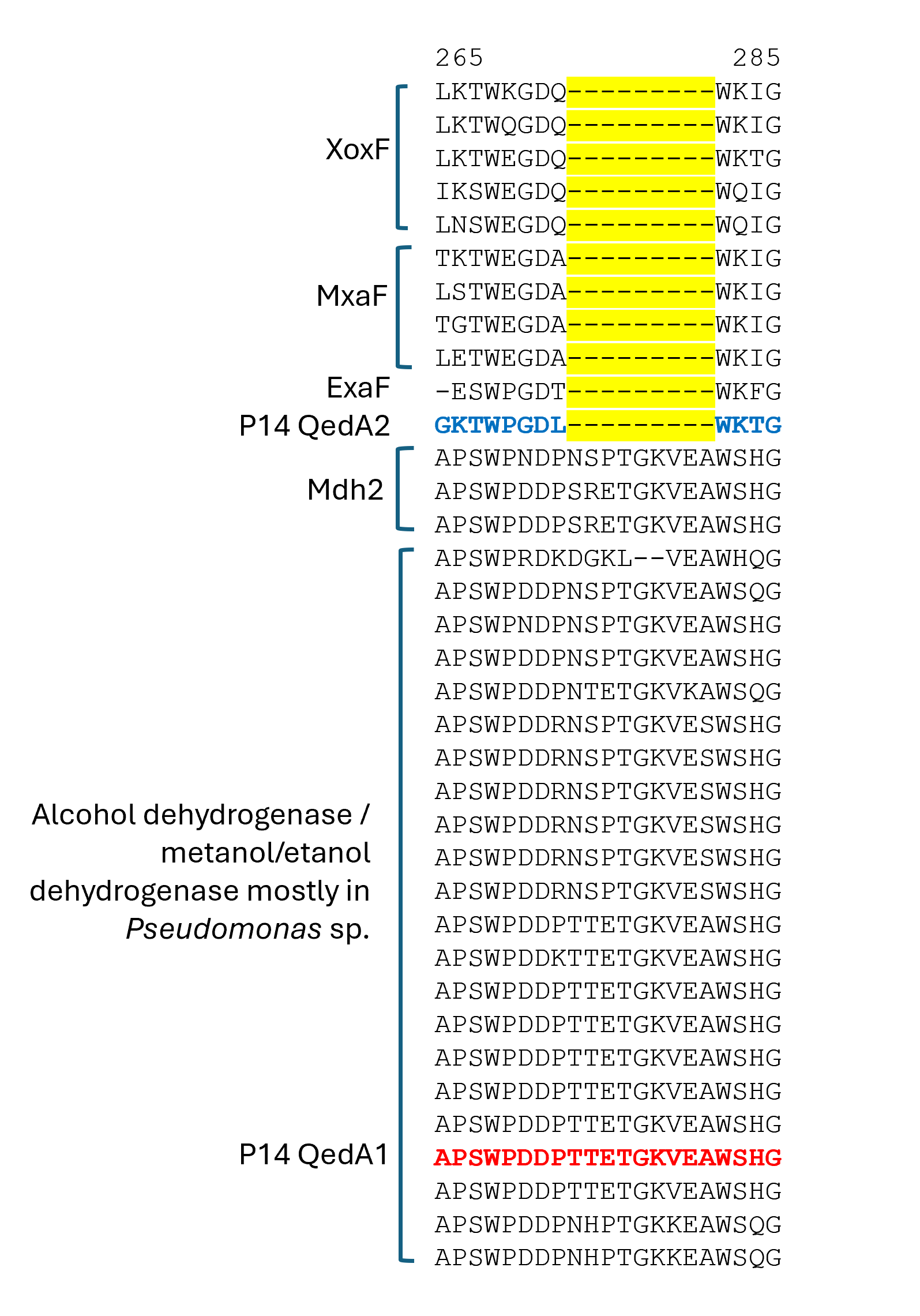

Supplement: Supplemental Information 6 [file peerj-14-20614-s006.png]

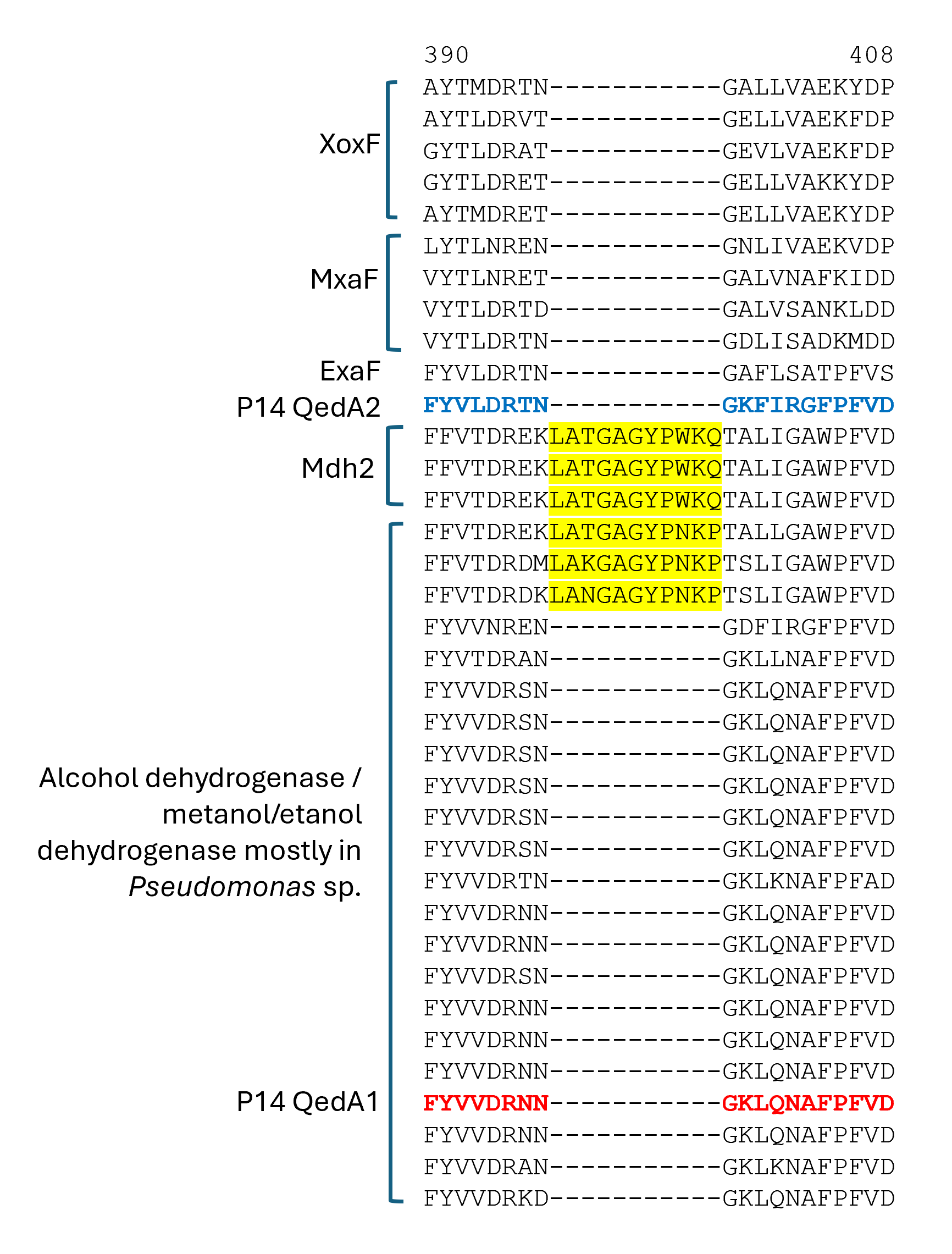

Supplement: Supplemental Information 7 [file peerj-14-20614-s007.png]

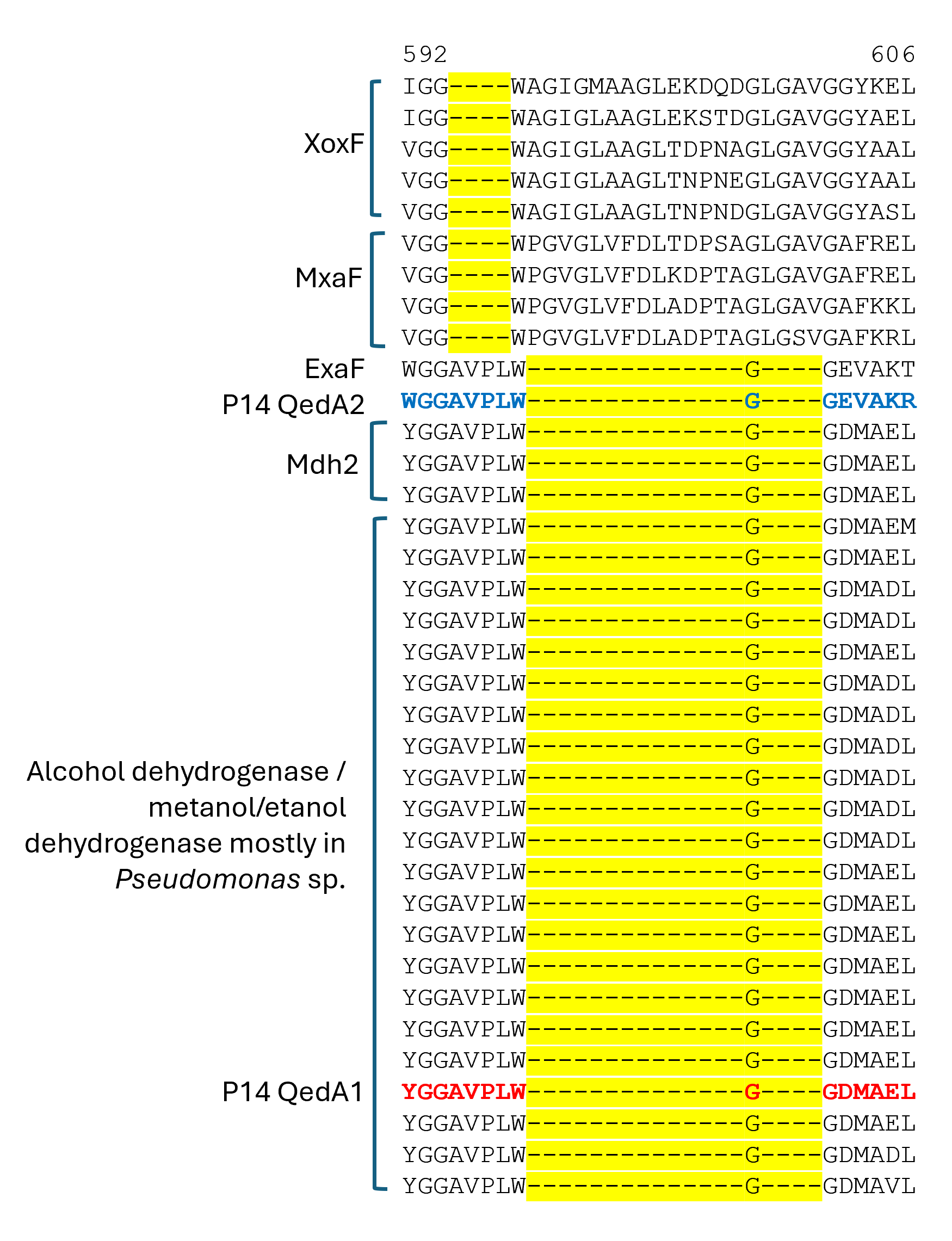

Supplement: Supplemental Information 8 [file peerj-14-20614-s008.png]
